# Sustained symptom reduction in complex regional pain syndrome with a novel home-based virtual reality program: a pilot study

**DOI:** 10.3389/fneur.2025.1622897

**Published:** 2025-12-08

**Authors:** Miles R. Fontenot, Michele Curatolo, Brett R. Stacey, Herta Flor, Hunter G. Hoffman

**Affiliations:** 1 Department of Anesthesiology and Pain Medicine, University of Washington, Seattle, WA, United States; 2 Department of Cognitive and Clinical Neuroscience, Central Institute of Mental Health, Medical Faculty Mannheim, Heidelberg University, Mannheim, Germany; 3 Human Photonics Lab, Mechanical Engineering, Center for Human Neuroscience, Department of Psychology, University of Washington, Seattle, WA, United States

**Keywords:** complex regional pain syndrome, virtual reality, home-based therapy, pain catastrophizing, central sensitization, self-management

## Abstract

**Background:**

Complex regional pain syndrome (CRPS) often leads to limb disuse, pain catastrophizing, and depression. While physical movement of CRPS affected limbs is essential for improvement, pain discourages movement. Home-based virtual reality (VR) interventions may reduce movement-related pain, improve adherence, and promote sustained symptom reduction.

**Methods:**

In this exploratory pilot study, seven patients with CRPS completed a 4-month home-based VR program integrating three treatment modules (1) embodied cyberhands VR physical movement treatment, (2) VR-mindfulness-based-stress-reduction, and (3) VR-pain-neuroscience-education. The primary outcomes were patient-reported global impressions of change in CRPS-related pain and physical ability. Secondary outcomes included central sensitization, cold sensitivity, depression, pain catastrophizing, and upper-limb function.

**Results:**

On the primary outcome global impression of change measures, six of seven patients reported sustained improvement in CRPS-related pain and physical ability, with three reporting being “much” or “very much” improved at 4 months. Gains were maintained at 1-year follow-up. Secondary outcomes showed significant improvements at 4 months: central sensitization (CSI: 46.7 ± 11.97 to 38.4 ± 11.53), cold pain threshold (27.12 °C ± 3.89 to 24.56 °C ± 5.56), depression (CES-D: 23.0 ± 11.27 to 15.6 ± 8.85), pain catastrophizing (PCS: 24.3 ± 11.06 to 15.1 ± 9.67), and upper-limb function (QuickDASH: 47.7 ± 20.43 to 34.8 ± 17.47). Improvements were sustained at 1-year follow-up. No serious adverse events occurred, and study adherence was 100%.

**Conclusion:**

Results provide preliminary evidence that a home-based VR program may produce sustained improvements in CRPS-related pain, physical ability, and associated symptoms. Larger randomized controlled trials are recommended.

**Clinical trial registration:**

## Introduction

Complex regional pain syndrome (CRPS) is one of the most severely painful chronic pain conditions in medicine. CRPS predominantly affects females, and typically develops in a limb after an injury, after surgery, or after a stroke. CRPS is marked by severe allodynia and hyperalgesia. In extreme cases, even a light feather touch can elicit intense pain. Because movement of the affected limb is painful, patients often avoid using it, leading to disuse, atrophy, loss of strength, impaired function, and maladaptive neuroplastic changes in brain regions controlling the limb (1–5). Prolonged disuse is thought to contribute to pathological cortical reorganization similar to that seen in phantom limb pain (6–8).

CRPS remains poorly understood, and no universally effective treatment currently exists. Proposed mechanisms include an abnormal response of the nervous system to an injury, central sensitization, and pathological cortical plasticity. Some patients respond to available interventions, but many patients try a number of treatments without enduring improvement (9, 10). Encouraging patients to use their painful extremity is essential for recovery (11, 12), and patients should begin movement therapy as soon as possible (9). Unfortunately, because physical movement of their injured limb is painful, CRPS patients avoid using their injured limb (e.g., avoid doing their traditional movement therapy homework exercises). Pain during physical therapy often leads to poor adherence and limited real-world treatment impact.

Immersive virtual reality (VR) has emerged as a promising tool for pain management. VR distraction, usually without embodied avatars, reliably reduces acute procedural pain while patients are wearing a headset (13–16) i.e., VR distraction alone. However, transient VR distraction alone is unlikely to produce lasting changes in persistent pain conditions (17).

Trost et al. (18) point out that during the past 10 years, there has been a big increase in the number of studies exploring how to use virtual reality to help chronic pain patients, beyond VR distraction alone. According to Trost, most chronic pain VR studies take advantage of virtual embodiment (patients illusion of ownership of the avatar, e.g., the virtual hand is my hand).

Early VR-CRPS studies demonstrated short-term reductions in pain using static or motionless avatars. For example, visual manipulations of virtual limbs (19–21) produced temporary analgesia during VR exposure. In another study, a “cyberglove” was used to simulate mirror therapy by allowing movement of a virtual affected hand controlled by the contralateral healthy hand, with some analgesic effects persisting after treatment (22, 23). While these pioneering studies highlight the potential of avatar-based VR, their benefits were largely short-term and often involved immobilized or passively manipulated avatars.

In contrast, recent randomized controlled trials in chronic low back pain have demonstrated long-term sustained benefits of home-based VR programs incorporating dynamic, movement-driven avatars (24–27). Embodied VR approaches, in which patients control virtual limbs, appear particularly promising for recalibrating pathological body representations and encouraging use of the affected extremity (28, 29). These findings suggest that multimodal VR interventions combining embodied avatar movement, psychological strategies, and education may support durable improvements. Building on this literature, the current study tests a novel home-based, multi-component VR program for patients with upper-limb CRPS, integrating three treatment elements: (1) embodied “cyberhand” movement therapy, (2) VR mindfulness-based stress reduction, and (3) VR pain neuroscience education.

The primary objectives of the current study were to assess patient-reported global impressions of post-treatment change in CRPS-related pain and physical ability. Secondary objectives were to evaluate post-treatment changes in central sensitization, depression, catastrophizing, and upper-limb function, and to examine whether improvements persisted at 1-year follow-up.

## Methods

### Overview

The current within-subject pilot study (*N* = 7) assessed a 4-month long, home-based, multi-modal VR program for upper-limb CRPS. The intervention combined three evidence-based VR treatments: embodied cyberhand movement therapy, VR-mindfulness-based stress reduction and VR-pain-neuroscience education.

Primary outcomes were assessed pre- versus post-treatment; and at 1-year follow-up.

### Participants and setting

Seven adults (≥18 years, age range 23–61 years old, mean = 46.45 years old, SD = 14.33 were studied. Five Caucasian females, one African American male and one Caucasian male) with CRPS I or II of the upper limb using Budapest Criteria (11, 30) and average pain ≥3/10 were recruited from the UW Center for Pain Relief. All were on stable medical regimens for ≥4 weeks, fluent in English, and capable of wearing an HMD. Exclusions: severe motion sickness, ASA ≥ IV systemic disease, or incarceration. Nine additional CRPS patients (seven female adults and two male adults) in the UW Roosevelt Clinic Registry were e-mailed via HIPAA compliant server to see if they were interested in participating in our study, but declined. The UW IRB approved the protocol (STUDY00015202 and STUDY00017399) and written informed consent was obtained per the Declaration of Helsinki. ClinicalTrials.gov ID NCT05888142. The study was conducted between September 2022 to July 2024.

### Study design

A pre–post within-subject design was used. Each patient served as their own control, (comparing symptoms before vs. after treatment) maximizing power in this small sample. All seven participants completed baseline assessments, 4 months of home-based VR therapy, and post-treatment assessments administered immediately before and after the 4-month treatment. Most patients returned for our *ad-hoc* 1-year follow-up questionnaires. Two patients could not be located for the 1-year follow-up measures. Intent-to-treat principles handled any missing follow-up data. Participants received a UW IRB/Ethics Committee approved compensation for their time and inconvenience.

### Intervention procedures

#### In-clinic training

This study tested whether the combination of commercially available VR programs (commercial cyberhand movement VR games and VR pain therapy modules) not designed specifically for treating CRPS, could serve as an intervention to treat CRPS. Participants attended 2–3 “in clinic” sessions to learn headset operation and software navigation. Each participant was loaned two HMD systems to use at home at the patient’s convenience: The Meta Quest 2 HMD (camera-based hand tracking) for movement therapy games and the Pico G2 4K HMD for mindfulness and Pain Neuroscience Education modules.

#### Home-based VR program

During the 4-month intervention, participants ideally self-administered daily VR “homeworks” using the following home-based VR treatments.

**Figure 1:**
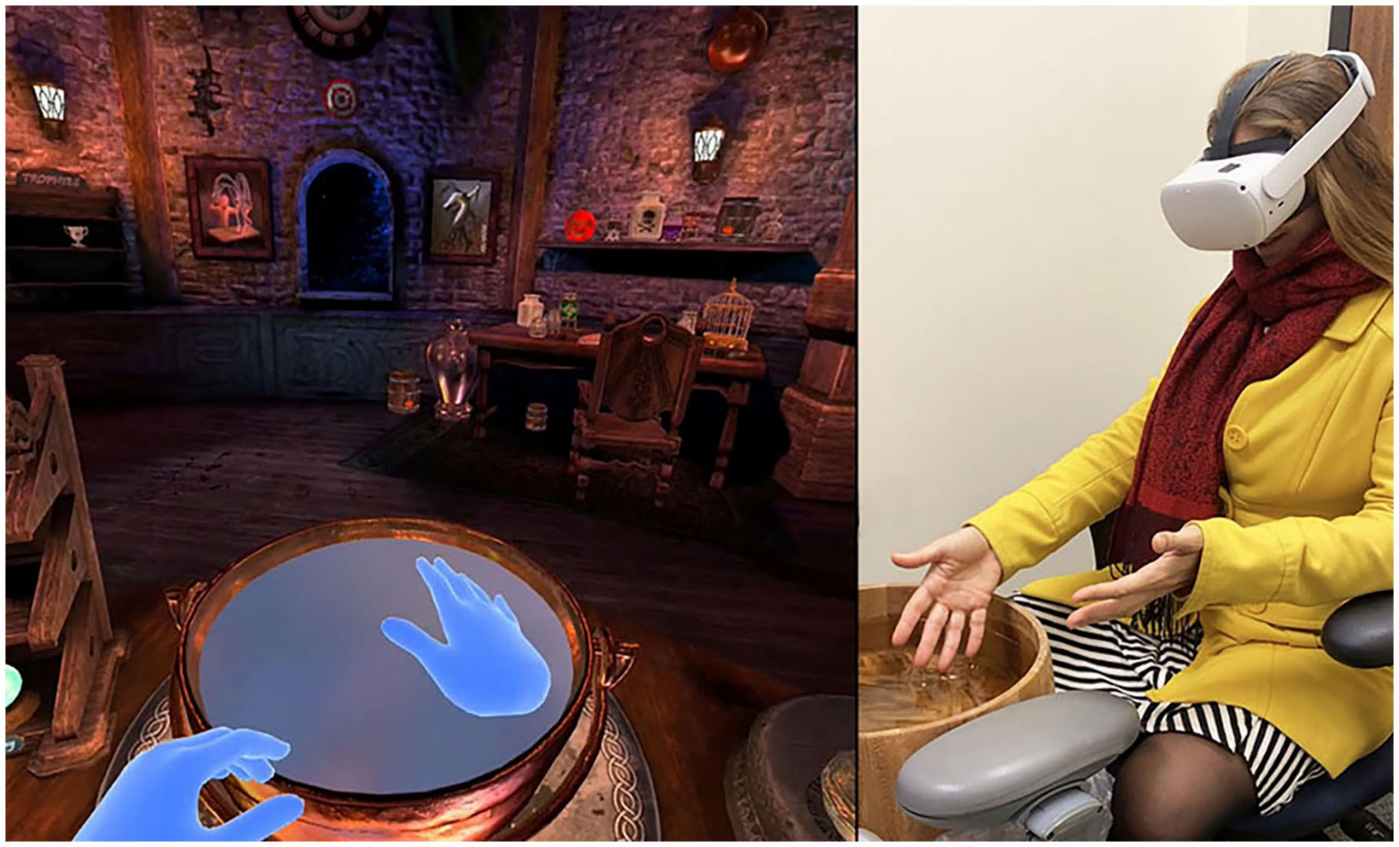
Embodied cyberhands VR movement therapy. Patients use camera tracked real hands to control “cyberhands” that grasp and place virtual objects. These repetitive, intuitive movements of the CRPS affected limb are intended to reduce brain atrophy from disuse and achieve sustained CRPS relief (The image on the left is from the Waltz of the Wizards by Aldin Dynamics, Reykjavik, Iceland, https://www.aldin.io/, used with written permission). Photo on right: copyright Hoffman, www.vrpain.com.

*Embodied cyberhand VR movement games* were used to drive embodied limb use (Figure 1). During VR cyberhand Movement Therapy, using an HMD with optical hand tracking capabilities, patients used their cyberhands (and, in turn, their real hands) to interact with virtual objects and to perform simple tasks requiring manual dexterity in the virtual world. These interactions with objects in the virtual world help motivate patients to physically move their affected limb. Patients were instructed to use their CRPS affected limb to manipulate virtual objects in the games. In other words, they were tasked to perform unimanual activity with their CRPS affected limb during home-based treatment.

Participants engaged in several commercially available interactive VR applications with cyberhands enabled obtained from the Meta Quest Store (Meta, Inc.). The titles included *Waltz of the Wizard* (Aldin Dynamics), *Hand Physics Lab* (Dennys Kuhnert – Holonautic; current app version 1.3.0.165), *The Curious Tale of the Stolen Pets* (Fast Travel Games), *Cubism* (Thomas Van Bouwel / Vanbo), and *Vacation Simulator* (Owlchemy Labs). All applications are available at the Meta.com Quest store, and were played on a Meta Quest 2 headset. VR games were selected for their accessibility, ease of use in home-based environments, low cost, and prior evidence of engagement and usability, calmness and use of camera-based hand tracking in non-clinical populations.

*CenteredVR mindfulness training* was developed as part of the Johns Hopkins Mindfulness Program. Using the Pico HMD, CenteredVR delivers a series of six 20 min mindfulness stress reduction sessions to help users bring their mind into the present moment, which can lower persistent stress, and the VR world includes educational elements and mindfulness practices that teach coping skills to help increase patients ability to manage their response to stressful situations so they become less reactive and more resilient to stressors in the real world. Stress often exacerbates chronic pain, reducing stress helps reduce chronic pain severity.

*VR pain-neuroscience-education (PNE) modules*. As summarized by (31) PNE 2.0 software (BehaVR Inc., Elizabethtown, KY, version 2.0) was delivered using a consumer grade PICO G2 4K VR head-mounted display (PICO Interactive, San Francisco, California, USA). PNE 2.0 is a 12-session VR-PNE program for chronic pain that uses both immersive real-world footage and interactive computer-generated imagery (CGI) to deliver visually and emotionally engaging education and relaxation training activities. VR-PNE combines traditional pain education modules, with customizable patient testimonials, and interactive emotion regulation practices such as breathing and guided mindfulness exercises in six different natural environments. The mean session time is approximately 21 min per session.

Participants could use the HMD at their own discretion. We encouraged participants to use VR for 15–20 min a day for 5 days a week. If they used it for longer than 20 min they were encouraged to take a brief break after 20 min.

### Outcome measures

#### Screening pre-treatment brief pain inventory (to make sure they were eligible for this study)

Primary endpoint (post-treatment at 4 months): Patient global impression of change (PGIC): Two 7-point scales (−3 to +3) for CRPS-related pain and physical ability (32).

Secondary measures (baseline and 4 months): Central sensitization inventory (CSI) (33, 34). Central sensitization refers to the amplification of nociceptive signals within the central nervous system, leading to increased sensitivity and pain even in the absence of ongoing tissue damage or a clear source of nociceptive input. The CSI is a well validated measure of variables related to central sensitization, and will be complemented by our quantitative sensory testing (QST) measures.

CES-D short form for depression (35, 36). Depressive symptoms was assessed with the Center for Epidemiologic Studies–Depression (CESD) Scale Short Form, also reported to have excellent psychometric qualities in past research (36). In the Burns et al., sample of chronic pain patients, the Cronbach’s alpha was 0.83 at the baseline assessment (37).

QuickDASH for upper-limb function (38, 39). The level of physical activity allowed despite limitations from the upper extremity with CRPS was assessed with the QuickDASH, which is an 11-item modified (shortened) version of the Disabilities Arm Shoulder and Hand (DASH) questionnaire.

PROMIS sleep disturbance–short form (40). Sleep disturbance was assessed with the 6-item Patient-Reported Outcomes Measurement Information System Sleep Disturbance Scale. It has shown excellent psychometric characteristics in past research (40).

Tampa Scale of Kinesiophobia (41). This scale measures patients “fear of movement” e.g., in patients with chronic musculoskeletal pain (42).

Pain Catastrophizing Scale (PCS) (32). This measures how often catastrophic thoughts related to pain occur. The PCS has been widely used in chronic pain and has excellent psychometric properties. According to a meta-analysis (43), the PCS has demonstrated good internal reliability (*α* = 0.92, 95% confidence interval 0.91–0.93) and test–retest reliability scores (Spearman *ρ* = 0.88, 95% confidence interval 0.83–0.93) were found for PCS total scores but not for subscales.

Single-item mindfulness GRS (0–10) following Kabat-Zinn’s definition of mindfulness, patients were asked “How mindful were you during the past week?” (on a zero to 10 graphic rating scale).

Simulator sickness was measured using a single item graphic rating scale (44). Participants were given instructions on how to minimize cybersickness. Furthermore, the VR worlds selected are calm by nature. All of the VR experiences had patients sitting down, with minimal head movements. Furthermore, the HMDs have minimal lens distortion, and high frame rate, which helps minimize simulator sickness. We did ask patients informally if they were experiencing simulator sickness during training, and via our single item graphic rating scale measure, and we also screened out people with a history of motion sickness.

#### Quantitative sensory testing (QST)

Thermal testing was performed using the Medoc Q-sense System™ (Ramat Yishai, Israel). Warm and cold detection thresholds, heat & cold pain thresholds, were measured via a Medoc Q-Sense thermode at baseline and 4 months. During the first quantitative sensory testing (QST) measurement session, the study team identified an area of the upper extremity that was affected by CRPS and made a detailed note of this location so it could be tested at the same location in future QST measurements. This anatomical location was not standardized and was specific to each subject’s presenting symptomatology. In other words, with the patient’s permission, the thermal stimulator was placed on (or near, in some cases) the body location with the most severe CRPS related sensitivity to pain. Patients were allowed to opt out of this measure and one patient did opt out of their CRPS QST measures. As a within-subject control, all QST measurements took place both on the ipsilateral upper extremity that is affected by CRPS and also on the equivalent anatomic location on the contralateral upper extremity that is not affected by CRPS.

Each QST measurement (e.g., cold pain threshold aka “just noticeable pain” to the CRPS affected hand) was repeated 3 times in a row, controlled by the Medoc computer program, and the mean of those three temperature measurements was used in the analyses.

At each time point, we used a comprehensive Quantitative Sensory Testing protocol based on (45), largely verbatim below. During testing, the computer screen was positioned so the participant could not view the laptop computer screen showing temperatures to the researcher. The participant was given a button to press when they first felt a change in temperature (during 3 brief stimuli), and also when they first felt a sensation of pain (during 3 more brief stimuli), and we recorded the temperature. The Medoc thermode has a contact area of 7.84 cm and was placed in contact with the participant’s skin. The Medoc software guided the examiner through a series of thermal testing procedures in the following order: cold detection threshold (CDT), warm detection threshold (WDT), cold pain threshold (CPT) i.e., “just noticeable pain,” and heat pain threshold (HPT). All thresholds were obtained with ramped stimuli (1 °C/s) that were terminated when the participant pressed the button connected to the Medoc Q-Sense. Cut-off temperatures are set at 16 °C (for cold pain) and 47 °C (for heat pain) with a baseline temperature of 32 °C.

One-year follow-up: All pen-and-paper measures were repeated after the 4-month treatment, and at 1 year follow-up. QST was measured at pre- vs. post-treatment but QST was not measured at the 1-year follow-up.

### Data analysis

Statistical analyses were conducted using repeated measures (pre-treatment vs. after the 4-month treatment) one-tailed paired Wilcoxon test using SPSS (version 29) software. We also conducted *post-hoc* Wilcoxon paired comparisons comparing pre-treatment vs. 1-year post-treatment measures. Standard deviation and standard error are reported. Missing 1-year data were addressed via last-observation-carried-forward, (or imputation for QST for one CRPS patient who did not have baseline QST data to carry forward for their CRPS QST measure) for intent-to-treat analyses. Statistical significance was set at *p* < 0.05.

### Participant flow and adherence

All seven enrolled CRPS patients (100%) completed baseline and 4-month post-treatment questionnaire assessments.

### Primary outcomes

#### Patient global impression of change (PGIC)

**Figure 2:**
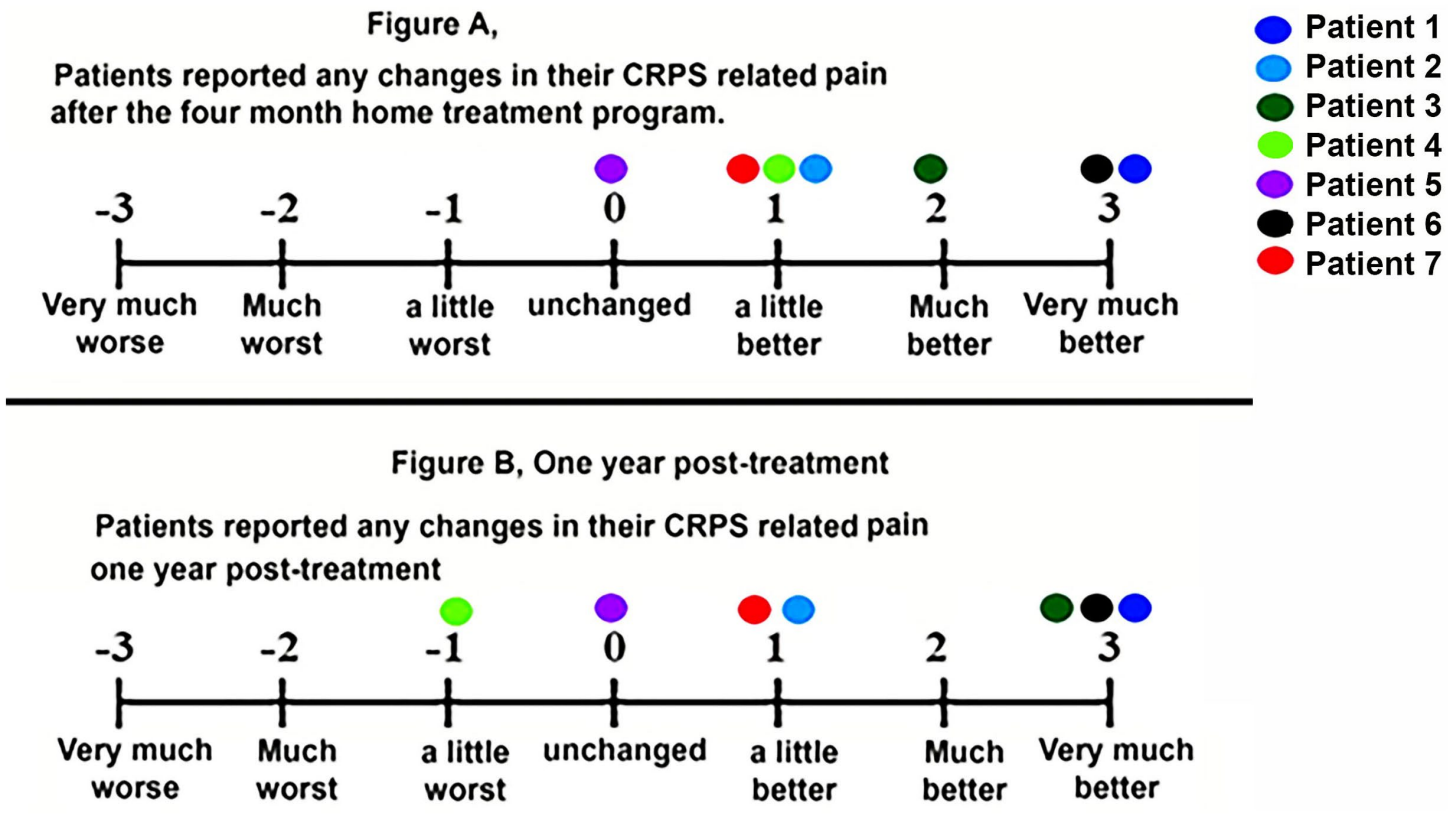
Changes in CRPS-related pain at post treatment **(A)** and at 1-year post-treatment **(B)**. Each of the seven patients has their own color.

**Figure 3:**
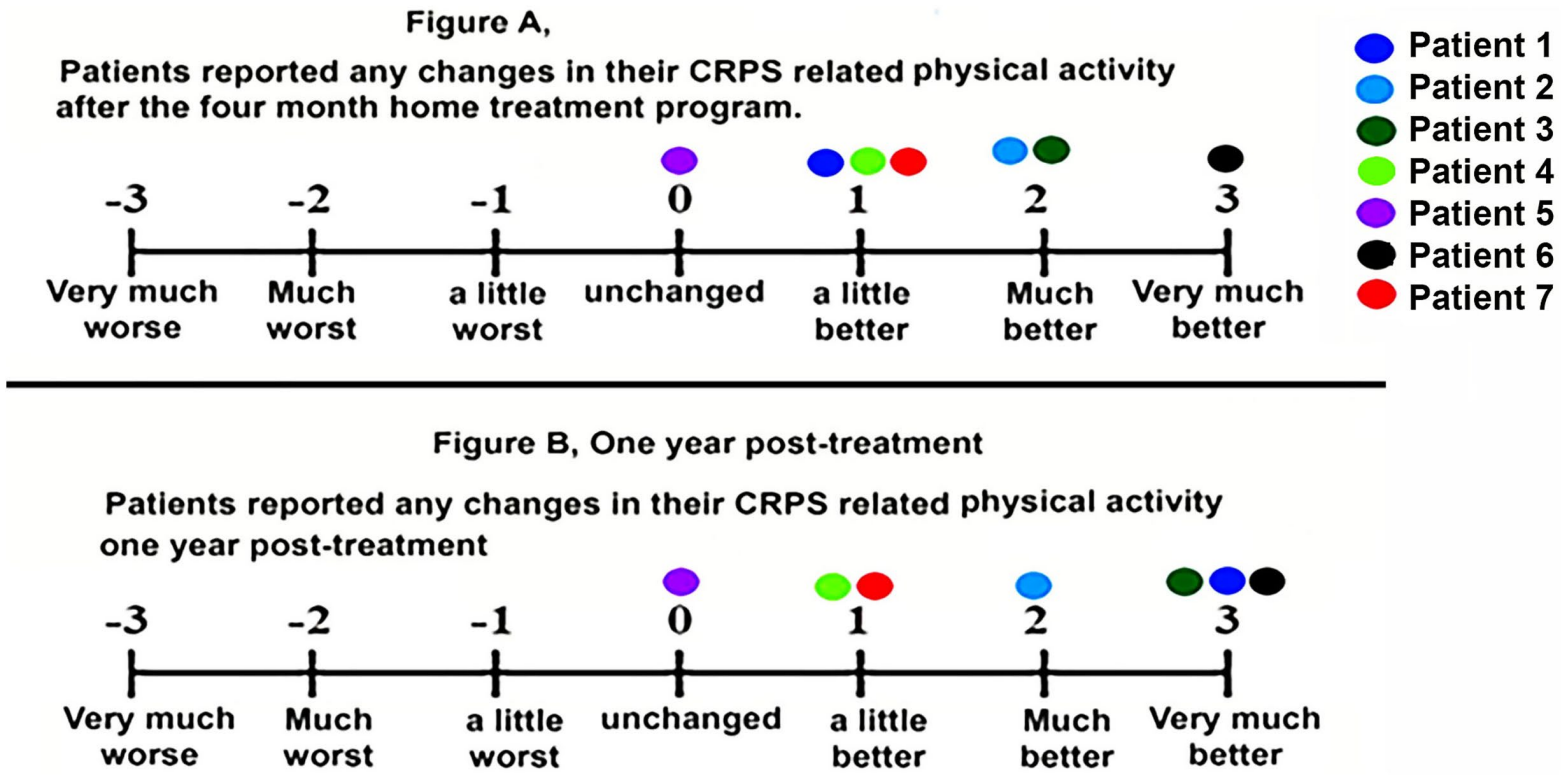
Changes in CRPS-related physical activity post treatment **(A)** and 1-year post-treatment **(B)**. Each of the seven patients has their own color.

At 4 months, six of seven participants reported overall improvement in CRPS-related pain and physical ability. Three patients rated themselves as “much” or “very much” improved. As shown in Figures 2, 3, these gains were maintained at 1-year follow-up in most patients.

As shown in Figure 2, on our primary outcome measure, most patients reported positive changes in their CRPS-related pain after the 4-month treatment, and these gains were maintained at 1-year follow-up.

Similarly, as shown in Figure 3, most patients reported positive change (i.e., improvement) in their CRPS-related physical activity after the 4-month home-based VR treatment, and these gains were maintained at 1-year follow-up.

### Secondary outcomes

#### Central sensitization

**Figure 4:**
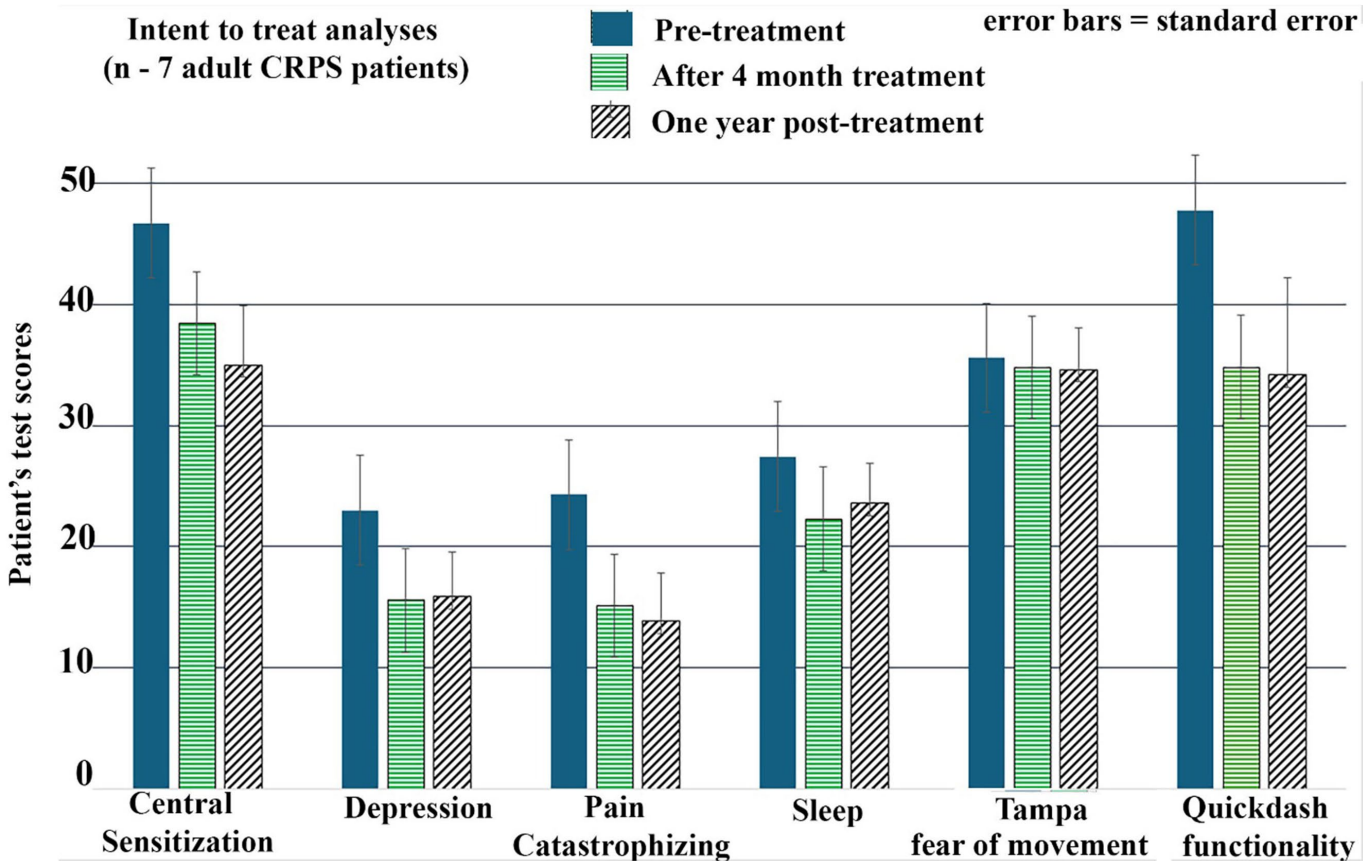
Patients’ ratings (means with standard error bars) on traditional pen and paper measures at baseline (solid) vs. after 4 months of VR treatment (horizontal stripes) vs. 1-year post-VR treatment (slanted stripes). The results show improvement: CRPS patients got better after completing the home-based VR treatment, and gains were maintained at 1-year post-treatment. Note that each measure has its own scoring calculation instructions.

The central sensitization inventory (CSI) improved significantly from baseline (46.7 ± 11.97) to post-treatment (38.4 ± 11.53; Z = 2.03, *p* < 0.05), with reductions maintained at 1 year (Table 1; Figure 4).

**Table 1:** Displays pre- and post-treatment questionnaire scores, non-parametric Wilcoxon Z-statistics, after the 4-month home based VR treatment.

Outcome measure	Pre-treatment Means (SD)	Post-treatment Means (SD)	Wilcoxon Z-values	One-tailed *p* values
Central sensitization	46.71 (SD = 11.97) SE = 4.52	38.43 (SD = 11.53) SE = 4.36	*Z* = 2.03	*p* < 0.05 * significant
Depression	23.00 (SD = 11.27) SE = 4.26	15.57 (SD = 8.85) SE = 3.34	*Z* = 2.37	*p* < 0.05* significant
Pain catastrophizing	24.29 (SD = 11.06) SE = 4.18	15.14 (SD = 9.67) SE = 3.65	*Z* = 2.04	*p* < 0.05 * significant
Sleep	27.43 (SD = 4.20) SE = 1.59	22.29 (SD = 8.86) SE = 3.35	*Z* = 1.69	*p* < 0.05*, significant
Tampa (fear of movement)	35.58 (SD = 7.65) SE = 3.12	34.75 (SD = 9.78) SE = 3.99	*Z* < 1	*p* = 0.43 NS
Mindfulness	5.81 (SD = 2.12) SE = 0.8	6.29 (SD = 2.43) SE = 0.92	*Z* < 1	*p* = 0.27 NS
Quickdash (lower scores show improved upper limb functionality)	47.74 (SD = 20.43) SE = 7.72	34.80 (SD = 17.47) SE = 6.60	*Z* = 2.20	*p* < 0.05* significant

#### Quantitative sensory testing (QST)

Cold pain threshold (CPT) of the CRPS-affected limb improved significantly, dropping from 27.1 °C (±3.89) to 24.6 °C (±5.56; *Z* = 1.75, *p* < 0.05), indicating reduced hypersensitivity to cold stimuli. No significant changes were observed for other QST measures (*p* > 0.05). QST was not repeated at 1 year.

#### Psychological symptoms

Depressive symptoms (CES-D) decreased significantly from 23.0 (±11.27) to 15.6 (±8.85, *Z* = 2.37, *p* < 0.05). Pain catastrophizing (PCS) decreased from 24.3 (±11.06) to 15.1 (±9.67; *Z* = 2.04, *p* < 0.05). Both improvements were sustained at 1 year (Table 1; Figure 4).

#### Upper-limb function

QuickDASH scores improved significantly (lower scores = better function) from 47.7 (±20.43) to 34.8 (±17.47; *Z* = 2.20, *p* < 0.05). Improvements were sustained at 1 year (Table 1; Figure 4).

#### Sleep, fear of movement, and mindfulness

Sleep disturbance scores improved at 4 months (27.4 ± 4.2 to 22.3 ± 8.9; *Z* = 1.69, *p* < 0.05), though gains were not significant at 1 year (*p* = 0.08). Fear of movement (Tampa Scale) and mindfulness scores did not change significantly at either time point (*p* > 0.05).

### Exploratory measures

#### Simulator sickness

Six participants reported no or only mild simulator sickness. One participant reported moderate nausea during training, which resolved within 20 min and did not interfere with study participation.

#### Home VR usage

As shown below, two participants tracked their VR usage. Patient 2 engaged extensively with the home VR treatment (2,995 total minutes; approximately 60% avatar movement therapy, 40% mindfulness/PNE) and reported sustained benefits. Patient 4 used VR less (1,324 min; primarily movement games) and demonstrated mixed outcomes.

##### Patient 2

The home-based VR activities recorded by patients two and four are as follows:

- Grand total number of minutes using home-based VR for Patient 2 = 2,995 min.
- Total number of minutes of home-based VR therapy in each VR world of Patient 2.
- Cubism = 1,150 min total.
- VR Mindfulness (Centered VR) + Pain Neuroscience Education = 1,195 min total.
- Hand Physics World = 200 min total.
- Curious Case of the Missing Pet 215 min total.
- Waltz of the Wizards = 160 min total.
- Vacation = 55 min total.
- As shown in Figures 2, 3, Patient 2 reported that her CRPS related pain was “a little better” after the 4-month treatment, and at 1-year post-treatment. Her CRPS-related physical activity was much better after the 4-month treatment and at 1-year post-treatment.

##### Patient 4

- Grand total number of minutes using home-based VR for Patient 4 = 1,324 total minutes.
- Cubism = 544 min total.
- VR mindfulness and pain neuroscience education = 40 min.
- Hand Physics Lab = 410 min total.
- Curious Case of the Missing Pet = 80 min.
- Waltz of the Wizards = 250 min total.
- Vacation = 0 min.

#### Qualitative feedback

Four patients reported strong satisfaction with VR therapy, noting increased motivation for limb use and reduced focus on pain. For example, one participant stated: *“When I am in VR, I move my hand because I want to complete the task. I’m using my hand more and not thinking about the pain.”* One participant reported no benefit.

Patients also showed improvements such as reduced co-morbid CRPS-related psychological symptoms on brief pen and paper questionnaires after 4 months of home-based multi-treatment VR therapy. As shown in Table 1 and Figure 4, compared to baseline, after the 4-month treatment, patients reported significant reductions in depression, significant reductions in catastrophizing, and a significant increase in their self-rated ability to perform activities involving upper limbs in everyday activities. These gains were sustained at 1-year post-treatment follow-up (see Table A1; Figure 4; Table 2). There was no significant long-term reduction in fear of movement, and no significant long-term improvement in mindfulness or sleep quality. A score of 37 or above on the Tampa Scale suggests the presence of clinically significant kinesiophobia, where fear of movement may be impacting daily activities and functional abilities, so since the mean was <37, over half of the patients did not have clinical severity of fear of movement at pre-treatment.

**Table 2:** Patients continued to show large persistent reductions in CRPS symptoms on the pen and paper psychological measures after the 4-month treatment, and on the 1-year post-treatment measures.

Outcome measure	After the 4 month VR treatment, 1 year post-treatment	1 year post-treatment (intent to treat analyses for 1 year analyses, Table A1)
Global CRPS related pain	43% much better	43% very much better
CRPS related physical ability	43% much better	57% much better
Central sensitization	*p* < 0.05 (all *p* = one-tailed)	*p* < 0.05
Depression	*p* < 0.05	*p* < 0.05
Pain catastrophizing	*p* < 0.05	*p* < 0.05
Sleep	*p* < 0.05	*p* = 0.08 NS
Fear of movement	*p* > 0.05 NS	*p* > 0.05 NS
Upper limb functionality	*p* < 0.05	*p* < 0.05

### Brief pain inventory (BPI)

Patients filled out the Brief Pain Inventory (BPI) before beginning treatment. On a scale from zero (no pain) to 10 (pain as bad as you can imagine), their pre-treatment rating of “average pain intensity” was 5.67 (minimum = 3, maximum = 7, SD = 1.37, *n* = 6). Four of the seven patient’s post-treatment BPI results are also available. On a zero to ten scale, the Pain severity score on the BPI was 5.44 pre-treatment vs. 4.75 post-treatment, and the Average Pain Interference Score on the BPI was 5.00 pre-treatment vs. 4.29 post-treatment (*n* = 4). Some patients did not receive this exploratory measure. Both BPI Pain Severity Scores and BPI Pain Interference Scores were lower after treatment than pre-treatment (where lower is better).

Other stable treatments received by 3 of the 7 patients in this study for which information is available are as follows:

- Patient 2. Memantine, LDN, pregablin, bupropion, vitamin c, tumeric, compound pain cream.
- Patient 4. Lyrica Tizanidine Baclofen Memantine Duloxetine Tylenol.
- Patient 6. Occupational therapy/Gabapentin.

In summary, most patients benefited from home VR therapy, but there were large variations in outcome.

## Discussion

This exploratory pilot study found that after 4 months of home-based, multi-modal VR therapy, CRPS patients demonstrated significant improvements on the primary outcome measures: global impression of change (CRPS related pain and CRPS related physical ability at post-treatment), with gains generally maintained at 1-year post-treatment. Secondary outcomes showed significant post-treatment improvements in central sensitization (CSI), cold pain threshold of the affected limb (QST), depressive symptoms (CES-D), pain catastrophizing (PCS), and upper-limb function (QuickDASH), and these improvements were maintained 1 year later. Sleep disturbance, kinesiophobia (Tampa) and mindfulness did not change significantly on long-term measures. No serious adverse events occurred and adherence was high.

### Targeting central sensitization and movement avoidance

The combination of embodied movement, mindfulness, and pain neuroscience education was designed to address central mechanisms (sensitization, maladaptive plasticity) and behavioral barriers (movement-evoked pain, avoidance). The reductions/improvements in both the subjective measure of central sensitization (CSI) and reductions in sensory hypersensitivity (lower cold pain threshold during brief cold thermal stimuli) suggest reduced central hyperexcitability after the intervention, consistent with prior work showing positive associations between the CSI and QST pain thresholds, e.g., (46). Embodied “cyberhand” interaction required goal-directed, uni-manual use of the affected limb, which may counter disuse and support adaptive reorganization (2, 3).

### Beyond distraction

Sustained improvements of persistent pain likely require more clinically sophisticated treatments than VR distraction alone (17). Achieving long-term sustained reductions in CRPS symptoms will likely require improvements in how the brain processes nociceptive information, (i.e., even when the patient is not wearing the HMD). The current study tested whether the combination of commercially available VR programs (commercial hand tracking enabled movement VR entertainment games with cyberhands) and also VR Mindfulness and VR Pain Neuroscience Education pain therapy modules (with no avatars) designed to treat a wide range of chronic pain syndromes, could serve as an intervention to treat CRPS. Home based embodied cyberhand VR Movement therapy is designed to stimulate the parts of the CRPS patient’s brain that control the injured limb (the CRPS patient’s sensory cortex and the motor cortex), and strengthen muscles in the injured limb (e.g., their hand). Home-based VR enables movement, and frequent VR movement sessions help reduce CRPS symptoms. In other words, VR increases physical movement, and the physical movement improves outcome.

CRPS is a challenging chronic pain syndrome, often exacerbated by comorbid psychological symptoms. Consistent with a bio-psycho-social model of pain, e.g., (47), the current study used a multi-treatment approach. In addition to the embodied cyberhand VR treatment encouraging physical movement treatment, the home-based psychological VR Mindfulness treatment and psychological VR Pain-Neuroscience-Education treatments used in the present study are designed to help calm the patient’s nervous system, to help reduce hypersensitivity to pain, and to help reduce co-morbid psychological issues (e.g., catastrophizing and depression) that can exacerbate chronic pain severity.

### Relation to prior work

Most of the previous studies using VR to treat CRPS have used static avatars. For example, the researcher posed the patient’s real arm on a real table such that when the patient put on their goggles, they saw a virtual arm in the same place as their real arm (co-located). In contrast, the current study uses camera-based dynamic embodied avatar cyberhands, which allows patients to move and use their real hands and fingers to control their cyberhand and cyberfinger movements. The “hands free” camera-based hand tracking human computer interface we used in the current study requires and encourages intuitive and natural therapeutic physical hand and finger movements (e.g., grasping movements). Avatar movement (e.g., via embodied cyberhands) is likely much more effective than the static avatars used in most previous VR CRPS studies (e.g., early studies co-localizing patients arm with a stationary virtual arm/hands).

The current VR study is designed to achieve long-term reductions in CRPS related pain. Several previous early pioneer CRPS studies by other researchers (often conducted “in clinic”) have recently shown encouraging short-term reductions in the pain of CRPS patients during VR experiences. Although valuable, most of the early pioneering VR CRPS studies to date have only measured short term reductions in how much CRPS pain the patients experienced while they were in virtual reality, and long term follow-up measures are rare in VR CRPS studies to date. In contrast, the current avatar movement VR study measures long-term reductions in CRPS related pain, and other long-term benefits. Our results extend earlier work by demonstrating sustained functional and psychological improvements, including improved movement, reduced pain sensitivity, and self-reported global change in both CRPS related pain and CRPS related physical activity.

### Wider applicability and technological promise

The current CRPS study used camera-based hand tracking with embodied cyberhands and cyberfingers in a home-based VR treatment for CRPS. Consistent with the current results (with CRPS patients), other recent home based VR therapies for other chronic pain populations are also showing significant benefits of home-based dynamic avatar movement VR treatments for lower back pain (24–27).

Until recently, opioids were commonly prescribed liberally for treating chronic pain, including CRPS, but in light of the current opioid overdose crisis, the Federal Government has greatly restricted prescriptions for opioid analgesics. The development of novel and effective non-opioid pain control techniques is an urgent national priority for all branches of medicine (17).

Chronic pain is one of the leading causes of hospital visits, and chronic pain causes an enormous economic burden in the United States, resulting in over a half a trillion dollars a year in annual hospital costs, lost income and reduced productivity (48). Thus, a new low-cost non-drug high technology analgesia technique/treatment that can help patients achieve long term reductions in persistent chronic pain levels (i.e., reduced pain during their everyday lives when patients are not in VR) would be a highly valuable, cost-saving and humanitarian medical advance with important clinical and economic implications (if larger RCTs replicate the current findings).

## Limitations

The current results should be interpreted cautiously, for several reasons. This was an exploratory study with small sample size (*N* = 7) prioritizing statistical power to detect true effects. Participants and investigators were unblinded, introducing expectancy bias. Usage data were incomplete for most participants, limiting dose–response analyses. Generalizability is restricted by recruitment from a single center and inclusion of English-speaking participants only. The current study should be replicated using a between-group design, ideally with participants blinded to VR treatment group conditions (49), using a much larger sample size, and concealed allocation of randomization to reduce selection bias.

## Future directions

Virtual reality is a rapidly improving medical technology with an emerging role in the treatment of medical conditions (29), and some futuristic medical researchers predict that Medical VR/XR/AI may eventually become a new branch of medicine (50). VR therapy offers a non-invasive and low-cost, scalable option with minimal to no adverse effects. The patient’s ability to do these VR treatments at home at their leisure is an important advantage over traditional “in clinic” treatments, and patients’ 24 h a day access to our home-based VR technique for several months at a time may improve outcome. In the USA, some hospitals are projected to close, or lay off staff during the next few years due to large reductions in federal funding (e.g., reduced Medicaid). Home based VR may prove especially valuable for patients having difficulties getting traditional “in clinic” physical/occupational therapy appointments (e.g., patients who do not have insurance). Self-treatment at home using XR technologies is new, and may prove to be a game changer.

For standard movement therapy (traditional physiotherapy) 8–12 week programs are usually effective. Previous multimodal VR interventions used 56 days [8 weeks, (26, 27)] or shorter treatment periods, e.g., (31). The 4-month treatment duration used in the current study is unusually long. Future research should explore whether shorter treatments used for home-based VR studies could also be effective for home-based VR treatments for upper limb CRPS.

The current study uses VR worlds that were not designed to be used to treat CRPS. Recent progress has been made using VR worlds custom designed to treat chronic back pain (24–27). Therapeutic VR worlds custom designed for treating CRPS are needed. But in the meantime, new embodied cyberhand VR worlds that enable camera-based hand tracking (e.g., conducting an orchestra in a new game named “Maestro”) are slowly being added to the library of inexpensive commercially available games that could potentially be used to treat CRPS.

The first published VR analgesia study (14) used a $90,000 USD VR system to distract burn patients during wound cleaning. A much better lightweight wireless VR system now costs as low as $299 USD per VR system, inexpensive enough to send home with patients. With billions of dollars being invested by big tech into virtual reality, augmented reality, mixed reality technologies and artificial intelligence, the Federal Governments reduction of billions of taxpayers’ dollars currently used on traditional chronic pain treatments (e.g., Medicaid), and the unusually high cost of medical care in the USA, encouraging cost-cutting innovations, there is growing potential for harnessing these new computer technologies to help treatment resistant CRPS (and a potentially a wide range of other chronic pain syndromes), in the future. Additional research and development is recommended (e.g., avatar VR software specifically designed to treat CRPS is needed).

## Clinical and public health implications

Given the pressing need for effective non-opioid strategies in chronic pain and barriers to frequent clinic visits, a low-cost, home-based VR approach that improves pain and function and is generally well-tolerated could be a valuable adjunct to standard care. If confirmed in carefully controlled, adequately powered RCTs, such programs could expand access to rehabilitation, support self-management, and potentially reduce downstream costs associated with disability and healthcare utilization.

## Data Availability

The datasets presented in this article are not readily available because the aggregated data supporting the findings of this study are available from the corresponding author upon reasonable request. Requests to access the datasets should be directed to hunthoff9@gmail.com.

## Appendix

**Table A1:** Pre-treatment vs. 1 year post-treatment responses on pen and paper questionnaires.

Outcome measure	Pre-treatment Means (SD)	Post-treatment Means (SD) One year post-treatment	Wilcoxon Z-values	One-tailed *p* values
Central sensitization	46.71 (SD = 11.97) SE = 4.52	35.00 (SD = 13.03) SE = 4.92	*Z* = 1.99	*p* < 0.05* significant
Depression	23.00 (SD = 11.27) SE = 4.26	15.86 (SD = 9.79) SE = 3.70	*Z* = 2.03	*p* < 0.05* significant
Pain catastrophizing	24.29 (SD = 11.06) SE = 4.18	13.86 (SD = 10.46) SE = 3.95	*Z* = 2.04	*p* < 0.05* significant
Sleep	27.43 (SD = 4.20) SE = 1.59	23.57 (SD = 8.64) SE = 3.26	*Z* = 1.44	*p* = 0.08 NS
Quickdash (lower scores show improved upper limb functionality)	47.74 (SD = 20.43) SE = 7.72	34.15 (SD = 21.14) SE = 7.99	*Z* = 2.20	*p* < 0.05* significant
Tampa (fear of movement)	35.58 (SD = 7.65) SE = 3.12	34.58 (SD = 8.46) SE = 3.46	*Z* < 1	*p* > 0.05, NS
